# The UNC-45 Chaperone Is Critical for Establishing Myosin-Based Myofibrillar Organization and Cardiac Contractility in the *Drosophila* Heart Model

**DOI:** 10.1371/journal.pone.0022579

**Published:** 2011-07-25

**Authors:** Girish C. Melkani, Rolf Bodmer, Karen Ocorr, Sanford I. Bernstein

**Affiliations:** 1 Department of Biology, Molecular Biology Institute and Heart Institute, San Diego State University, San Diego, California, United States of America; 2 Development and Aging Program, Sanford-Burnham Institute for Medical Research, La Jolla, California, United States of America; University of Arkansas for Medical Sciences, United States of America

## Abstract

UNC-45 is a UCS (UNC-45/CRO1/She4P) class chaperone necessary for myosin folding and/or accumulation, but its requirement for maintaining cardiac contractility has not been explored. Given the prevalence of myosin mutations in eliciting cardiomyopathy, chaperones like UNC-45 are likely to be equally critical in provoking or modulating myosin-associated cardiomyopathy. Here, we used the *Drosophila* heart model to examine its role in cardiac physiology, in conjunction with RNAi-mediated gene silencing specifically in the heart *in vivo*. Analysis of cardiac physiology was carried out using high-speed video recording in conjunction with movement analysis algorithms. *unc-45* knockdown resulted in severely compromised cardiac function in adults as evidenced by prolonged diastolic and systolic intervals, and increased incidence of arrhythmias and extreme dilation; the latter was accompanied by a significant reduction in muscle contractility. Structural analysis showed reduced myofibrils, myofibrillar disarray, and greatly decreased cardiac myosin accumulation. Cardiac *unc-45* silencing also dramatically reduced life-span. In contrast, third instar larval and young pupal hearts showed mild cardiac abnormalities, as severe cardiac defects only developed during metamorphosis. Furthermore, cardiac *unc-45* silencing in the adult heart (after metamorphosis) led to less severe phenotypes. This suggests that UNC-45 is mostly required for myosin accumulation/folding during remodeling of the forming adult heart. The cardiac defects, myosin deficit and decreased life-span in flies upon heart-specific *unc-45* knockdown were significantly rescued by UNC-45 over-expression. Our results are the first to demonstrate a cardiac-specific requirement of a chaperone in *Drosophila*, suggestive of a critical role of UNC-45 in cardiomyopathies, including those associated with unfolded proteins in the failing human heart. The dilated cardiomyopathy phenotype associated with UNC-45 deficiency is mimicked by myosin knockdown suggesting that UNC-45 plays a crucial role in stabilizing myosin and possibly preventing human cardiomyopathies associated with functional deficiencies of myosin.

## Introduction

The myosin superfamily consists of at least 35 classes of actin-based molecular motors. These motors are critical for cellular processes such as cytokinesis, vesicle transport, cell motility, and muscle movement [1]. Muscle myosin II (referred to hereafter as myosin) is a major component of muscle thick filaments and thus indispensable for muscle contraction [2]. Compromised myosin function due to its mutation and/or deficiency has been associated with several human diseases including cardiomyopathy and skeletal muscle myopathy [3]–[10]. Chaperones are involved with multiple pathways including myosin folding and are critically required for maintaining cardiac function [11]–[14]. A growing body of evidence suggests that folding and stability of myosin is dependent upon UNC-45 levels [15]–[22]. UNC-45 functions as a chaperone and as a co-chaperone for HSP-90 *in vitro* [20], [21], [23]. UNC-45 is present in both invertebrates and vertebrates and is important for myosin maturation, thick filament assembly and muscle function [18], [24]–[26].

Structurally, UNC-45 is composed of three domains. The N-terminal tetratricopeptide repeat (TPR) domain is necessary for binding to HSP-90 [20]. The unique central domain is of unknown function and contains limited homology with β-catenin, a protein associated with the wingless signaling pathway [23]. The C-terminal UCS (UNC-45/CRO1/She4P) domain of UNC-45 is homologous to domains of fungal CRO1 and yeast She4p proteins; all associate with many classes of myosin [27]-[31]. Recently X-ray crystal structures of *Drosophila* UNC-45 and the yeast UCS protein She4p have been resolved; these proteins mainly consist of armadillo-like helical repeats [27], [31]. C. *elegans* and *Drosophila* each contain a single *unc-45* gene. *C. elegans unc-45* mutants are characterized by uncoordinated movement and abnormal muscle structure [16]. In *Drosophila* UNC-45 possesses chaperone activity that is required for myosin accumulation, and a null mutation leads to embryonic lethality [17], [21]. Unlike *C. elegans* and *Drosophila*, vertebrates have two *unc-45* genes [24], [26]. One is expressed predominantly in cardiac and skeletal muscle and is known as *UNC-45B*, whereas the other is expressed in multiple cell types and is designated *UNC-45A* [24]. In zebrafish and *Xenopus*, *UNC-45B* is required for cardiac and skeletal muscle function and a deficit or mutation leads to striated muscle dysfunction, paralysis and embryonic lethality [22], [25], [32], [33]. The UNC-45A isoform is involved in smooth muscle myosin maturation and in the development of the aortic arches [34], [35]. It appears that abnormal accumulation of UNC-45 is observed in a human inclusion body myopathy [36].

Although cardiac dysfunction and lethality are associated with inhibition of expression of the UNC-45B isoform [22], [25], [32], [33], UNC-45 function has not been explored at later stages and in adult specimens. This is essential for understanding UNC-45′s role in maintaining contractile force and preventing myosin-based cardiomyopathy. In this communication we use the *Drosophila* model to investigate UNC-45 function during metamorphosis and in adult cardiac tissue. *Drosophila* is an advantageous model for studying cardiac development and function since, unlike vertebrates, heart function can be significantly compromised without causing immediate death [37]. In addition, this system previously has been used as a model for human cardiac channelopathy and myopathy [8], [38]–[41]. The *Drosophila* heart is a tubular structure consisting of a single layer of contractile cardiomyocytes and non-contractile pericardial cells that align along each side of the heart wall. The heart is supported by alary muscles and, in adults, by a layer of ventral longitudinal muscle cells [37], [42], [43]. In the larva, the heart is divided into an anterior aorta and posterior heart. During metamorphosis, the posterior portion of the larval aorta is remodeled to form the adult heart [42]–[44]. During this process, a conical chamber (CC) is formed *de novo* in the first and second abdominal segments [42], [43] and three pairs of valves are formed in abdominal segments A2 to A4 [43], [44]. Heart segment A5 is transformed into a terminal chamber and A6 and A7 segments are removed by programmed cell death which is under hormonal control [42]–[45].

Here, we describe the use of *Drosophila* to study the establishment and maintenance of cardiac function associated with a myosin chaperone encoded by *unc-45*. Our genetic, structural and functional approaches demonstrate that UNC-45 is crucial for cardiac morphology, physiology and myosin accumulation in the myocardium. We show that *unc-45* silencing in third instar larval and young pupal hearts results in mild cardiac abnormalities. However, major cardiac defects appear as a result of knockdown during metamorphosis, indicating that UNC-45 participates in the process of remodeling of the adult heart to ensure normal sarcomeric structures and contractility. Cardiac *unc-45* silencing in the adult heart (after metamorphosis) leads to a less severe phenotype. Our demonstration that UNC-45 deficiency is mimicked by myosin knockdown suggests that UNC-45 plays a crucial role in stabilizing myosin and is likely involved in preventing human cardiomyopathy.

## Results

### Silencing of *unc-45* in the heart and its impact on cardiac function

**Figure 1:**
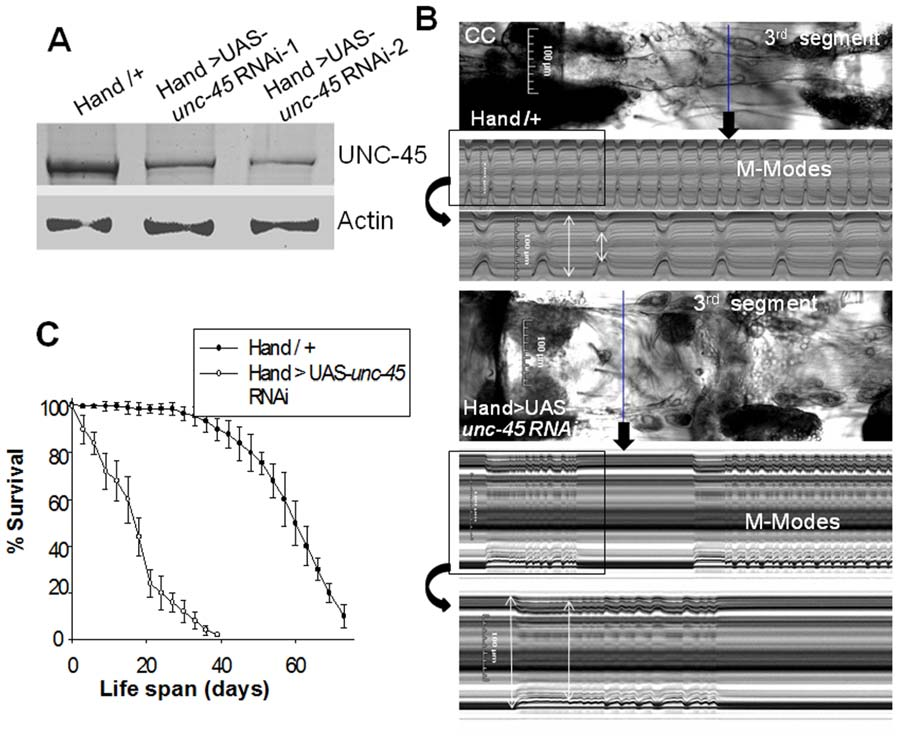
Cardiac defects associated with KD of *unc-45*. (**A**) Immunoblot analysis of UNC-45 expression in hearts from control (*Hand*/+) and *unc-45* KD (*Hand*>UAS-*unc-45* RNAi-1 (NIG) and *Hand*> UAS-*unc-45* RNAi-2 (Vienna *Drosophila* RNAi Center) flies. UNC-45 expression was reduced significantly (∼80%) in the KD hearts; however, actin expression remained unchanged. (**B**) Images of semi-intact conical chamber (CC) and cardiac tubes in third abdominal segments during systole and M-mode records from 1 week old control and *unc-45* KD flies. KD hearts showed significant dilation in the third abdominal segment of the cardiac tube, which was also apparent in M-mode analysis. M-mode records show heart wall movements over 15 and 5 (inset) sec time periods. Double-headed arrows in the M-mode traces indicate diastolic and systolic distances between heart walls. In addition to dilation, *unc-45* KD hearts developed arrhythmias (prolonged beating and episodes of fibrillation) whereas control hearts did not. (**C**) Cardiac-specific KD of *unc-45* directly impacted the life span of the flies; graph plots % survival (n = 250 for each group) vs. time post-eclosion.

To identify the role of UNC-45 in establishing normal heart function, we used an RNAi knockdown (KD) approach. Silencing of *unc-45* in the *Drosophila* heart was carried out using the UAS-Gal4 system [46]; the cardiac-specific *Hand*-Gal4 driver [47] was combined with UAS-*unc-45*-RNAi transgenes [48]. As shown in Figure 1A, *Hand*-Gal4 mediated KD of *unc-45* in hearts of 1 week old flies (*Hand*>UAS-*unc-45*-RNAi) reduced levels of UNC-45 by ∼80% compared to age-matched controls (*Hand*-Gal4/+). Analysis of heart function in 1 week old *Hand*>UAS-*unc-45* RNAi flies (referred to hereafter as *unc-45* KD) revealed severe heart defects compared to controls (Figure 1B, Movie S1): *unc-45* KD hearts were significantly dilated in both the CC and the third abdominal segment heart and exhibited dramatically arrhythmic beating patterns, compared to control hearts. Cardiac *unc-45* KD also had a drastic impact on the flies' life-span (Figure 1C). Interestingly, *unc-45* KD in all muscles using the mesodermal driver 24B-Gal4 [46] resulted in early developmental lethality and a dramatic reduction in the amount of myosin in the embryo was observed (Figure S1). This confirms our previous finding that *unc-45* null mutants were embryonic lethal and deficient in myosin accumulation [17].

### Cardiac physiological defects associated with *unc-45* KD

**Figure 2:**
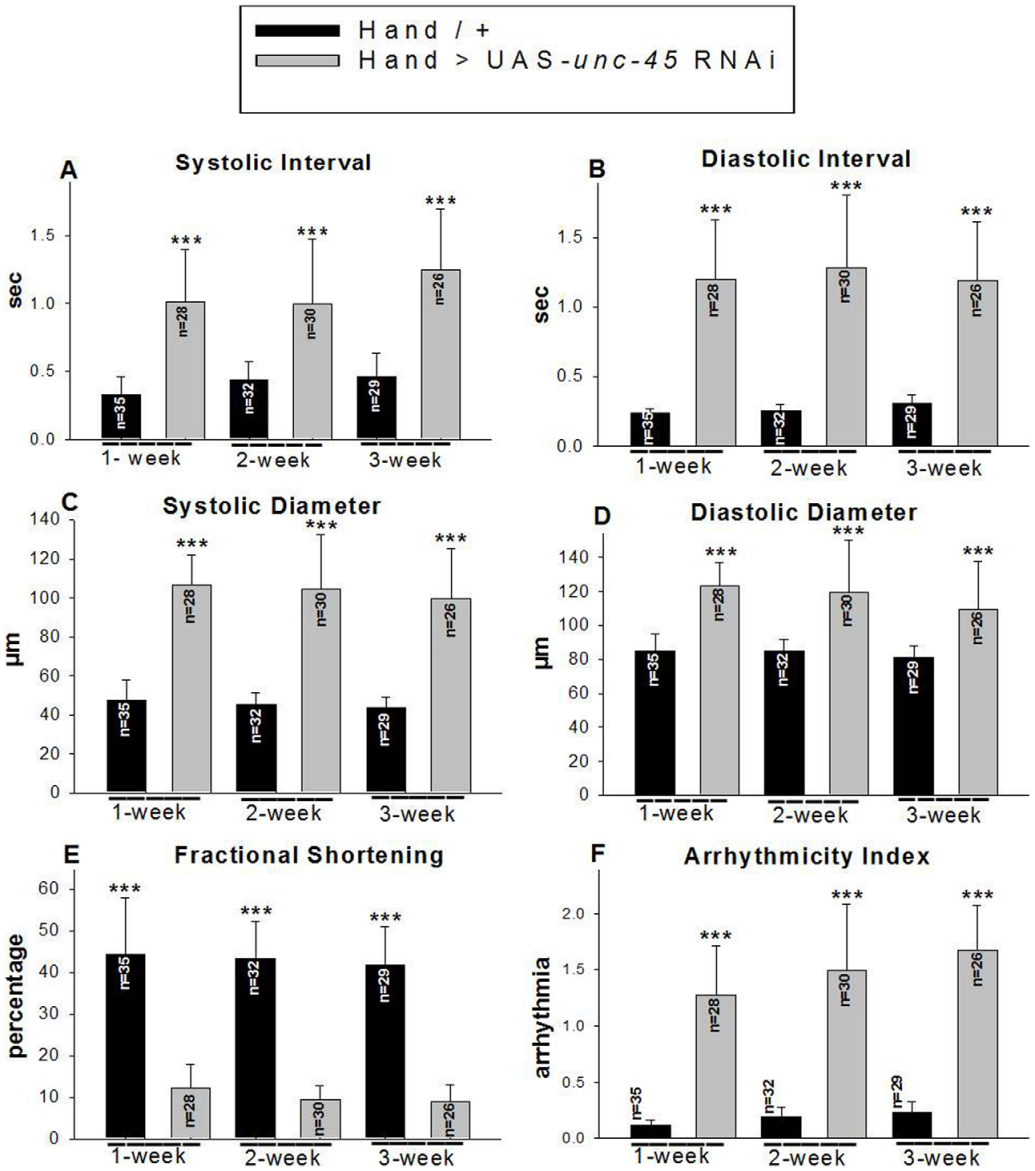
Physiological cardiac defects associated with *unc-45* KD. Cardiac parameters from the region between the second and third abdominal segments of the heart were determined as described in Materials and Methods. (**A, B**) Hearts from 1, 2 and 3 week old *unc-45* KD flies show prolonged systolic and diastolic intervals compared to control hearts. (**C, D**) Systolic and diastolic diameters of the KD hearts were significantly higher compared to those of age-matched controls. (**E**) Contractility (% FS) of the *unc-45* KD hearts was significantly reduced at all ages (**F**) Significant cardiac arrhythmias were observed in unc-45 KD hearts (quantified as arrhythmia index, see Materials and Methods). Data are shown as means ± SD; statistical significance was determined using a multivariate Student's t test (***  = p<0.001).

Quantitative analysis of various cardiac physiological parameters in 1-3 week old fly hearts revealed that KD of *unc-45* caused a significant prolongation of the heartbeat length, as manifested in an increase of both systolic and diastolic intervals (Figure 2A,B). Particularly dramatic is the cardiac dilation (Figure 1B), due to both systolic and diastolic heart diameter increases in *unc-45* KD hearts compared to control hearts (Figure 2C,D). The observed extreme cardiac dilation was accompanied by a significant reduction in heart contractility, which was quantified in a decreased fractional shortening (% FS; Figure 2E). Not only did we observe dilation and contractility changes in *unc-45* KD hearts, but the incidence of arrhythmias was also much higher in *unc-45* KD hearts compared to controls (the Arrhythmicity Index was increased 5–10-fold, even at very young ages) (Figure 2F). This was in large part due to the frequent occurrence of asystolies, intermittent stoppages in heartbeat (see Figure 1B, bottom M-mode traces).

### Structural dysfunction in *unc-45* KD hearts due to impaired myosin accumulation

**Figure 3:**
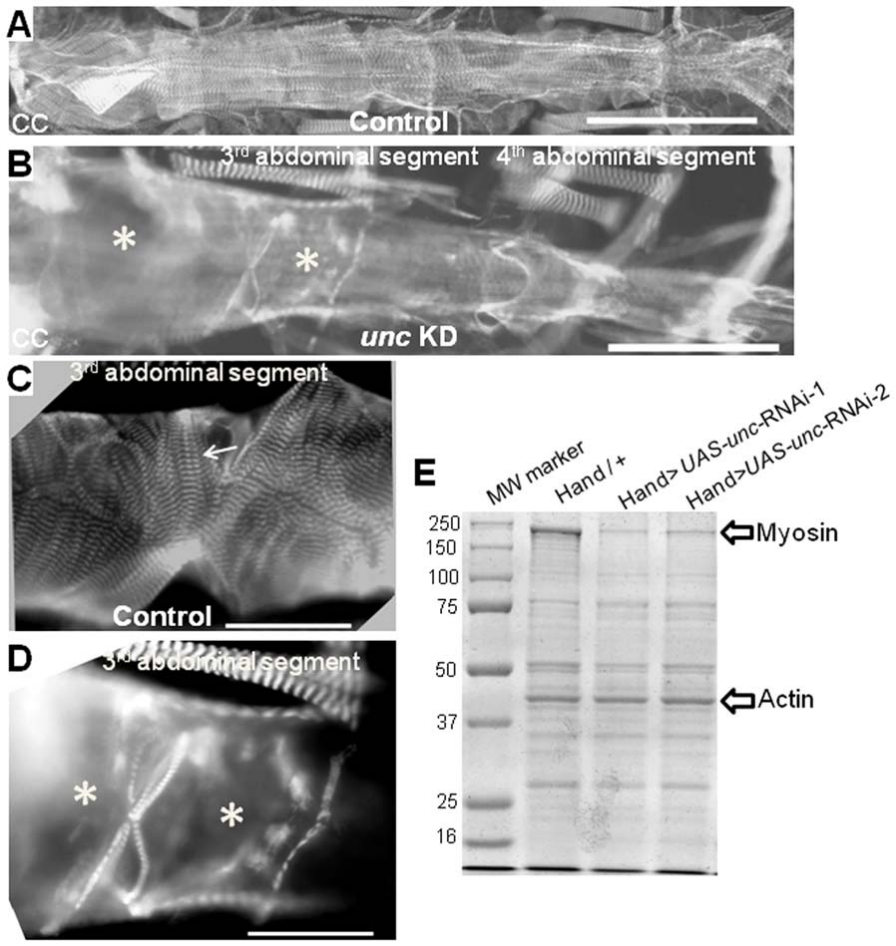
Structural dysfunction in *unc-45* KD hearts due to impaired myosin accumulation. Immunofluorescence micrographs of 1 week old flies are shown. (**A, C**) Hearts from controls and (**B, D**) *unc-45* KD flies were probed with antibody against muscle myosin. Control cardiac tubes show typical spiral myofibrillar arrangements within the cardiomyocytes (arrow). Myofibrillar organization is completely disrupted in the conical chamber and third segment of *unc-45* KD cardiac tubes (indicated by *) with loss of most myosin-containing myofibrils and significant dilation. Enlarged image (**D**) of the third abdominal segment of an *unc-45* KD heart demonstrates only a few myofibrils and gaps (missing myofibrils, indicated by *). Scale bars: 100 µM in A–B and 75 µM in C–D. (**E**) Myosin expression is significantly reduced in 1 week old *unc-45* KD hearts, however, actin expression remain unchanged as shown by SDS-PAGE.

To explore structural dysfunction associated with cardiac-specific KD of *unc-45*, control and KD hearts were probed with an antibody against myosin. Myofibrillar disarray was assessed with immunofluorescence microscopy. As shown in Figure 3A and C, control hearts are made up of densely packed myosin-containing myofibrils within cardiomyocytes. However, hearts from 1 week old *unc-45* KD hearts showed severe reduction in myosin content (Figure 3B and D), which resulted in myofibrillar disarray. Under high magnification, myocardial cells from control hearts exhibit a spiral arrangement of myosin-containing myofibrils (Figure 3C); however, the fibrillar myosin pattern was nearly completely lost upon KD of *unc-45* (Figure 3D). Consistent with the data obtained from live beating hearts, cardiac dilation was also seen in the fixed tissue from KD hearts (Figure 3B, D). Myofibrillar disorganization was also apparent when *unc-45* KD hearts were probed with phalloidin to visualize the actin organization within the myofibrils (Figure S2D) compared to wild-type control hearts (Figure S2B). Remarkably, even with minimal myosin present, myofibrils still form, albeit in a considerably disorganized fashion, and leading to extreme cardiac dilation and compromised contractility.

The lack of patterned myosin immunoreactivity in the myofibrils of *unc-45* KD cardiomyocytes showed a decrease in overall myosin. To further explore the mechanism underlying the effects of *unc-45* KD in the heart, we examined the impact of KD on myosin accumulation. UNC-45 is a chaperone that has been shown to be essential for myosin accumulation in muscle types other than heart (17, 22, 32, 33). As shown in Fig. 3E, myosin levels were drastically reduced (70–80%) in *unc-45* KD hearts compared to those in control hearts. However, actin expression remained unchanged. This is consistent with our observation with intact hearts probed with anti-myosin and phalloidin upon *unc-45* KD (Figure S2). The reduced myosin accumulation in *unc-45* KD hearts was confirmed by probing an immunoblot with myosin antibody (not shown). However, myosin expression in indirect flight muscles was similar in both control and *unc-45* KD flies (not shown) confirming that the KD was specific to heart muscle.

### *Mhc* KD mimics the *unc-45* KD cardiac phenotype

**Figure 4:**
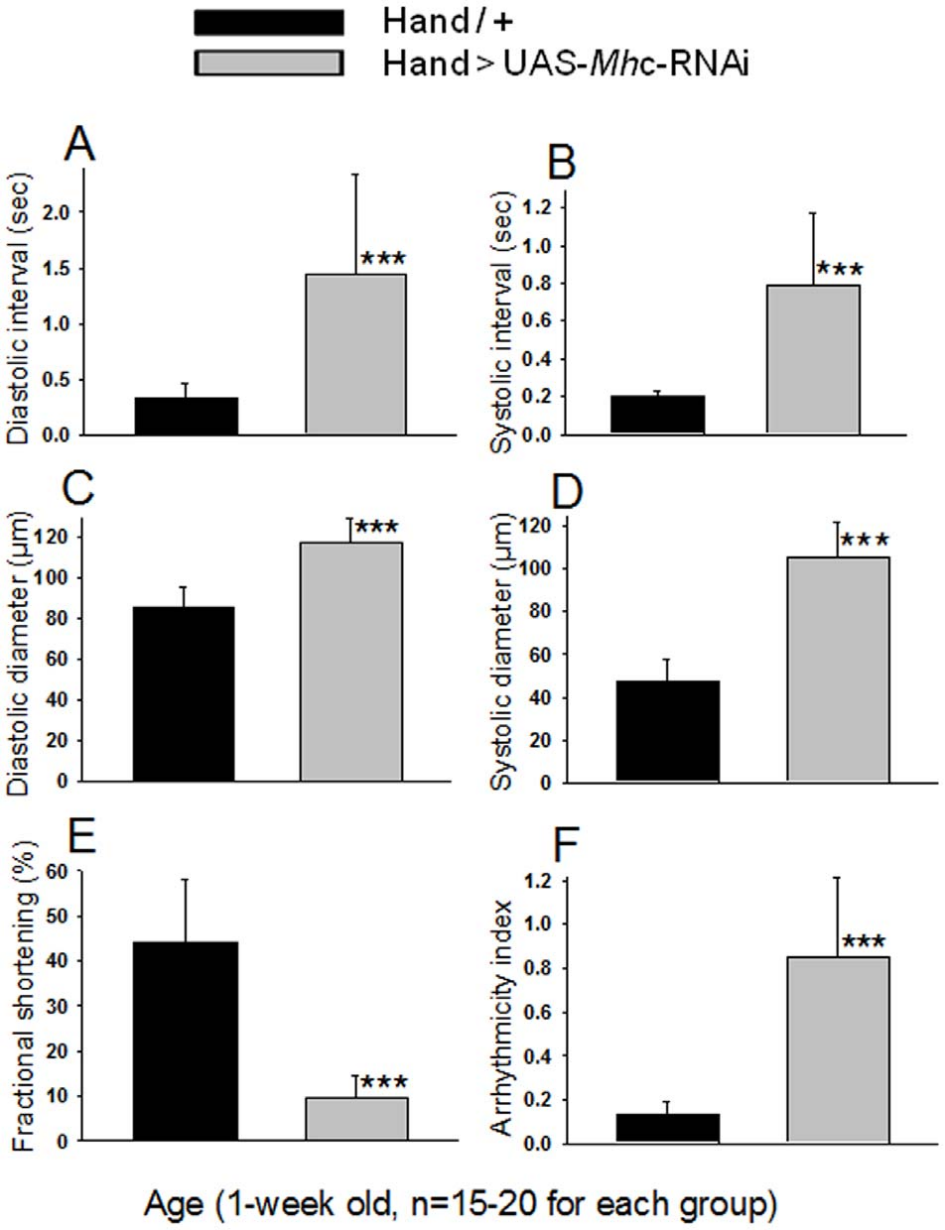
*Mhc* KD mimics the *unc-45* KD cardiac phenotype. Cardiac physiological parameters in hearts from 1 week old *Mhc* KD flies. (**A, B**) systolic and diastolic intervals were prolonged, (**C, D**) diastolic and systolic cardiac diameters were increased, (**E**) % FS was decreased (indicating reduced cardiac efficiency) and (**F**) cardiac arrhythmias were increased compared to age-matched controls. Data are shown as means ± SD; statistical significance was determined using a multivariate Student's t test (***  = p<0.001).

The decrease in myosin levels due to *unc-*45 KD suggested that the ensuing heart structure and function defects were mainly the result of myosin deficiency. Therefore, we tested if KD of myosin heavy chain (*Mhc)* itself can reproduce an *unc-45* KD-like phenotype. We evaluated flies with cardiac-specific *Mhc* KD using the *Hand-Gal4* driver. As for cardiac *unc-45* silencing, *Mhc* KD hearts showed prolonged systolic and diastolic intervals (Figure 4A,B) and increased diastolic and in particular systolic diameters (Figure 4C,D) resulting in reduced cardiac contractility (measured as % FS; Figure 4E). In addition, these hearts displayed a dramatic increase in cardiac arrhythmia, again due to increased asystoly events (Figure 4F). In addition to physiological cardiac defects, *Mhc* inhibition resulted in reduced accumulation of myosin and structural defects within the cardiomyocytes, in that direct inhibition of myosin abundance caused a similar disarray in myofibrils to that observed with cardiac *unc-45* KD (not shown). The finding that cardiac *Mhc* or *unc-45* KD produce a similar reduction in myosin abundance as well as similar cardiac structure/function phenotypes suggests that UNC-45 may be not only critical for myosin accumulation in the myocardium but also for establishing or maintaining normal myosin-dependent cardiac functionality.

### *unc-45* functions during cardiac metamorphosis

**Figure 5:**
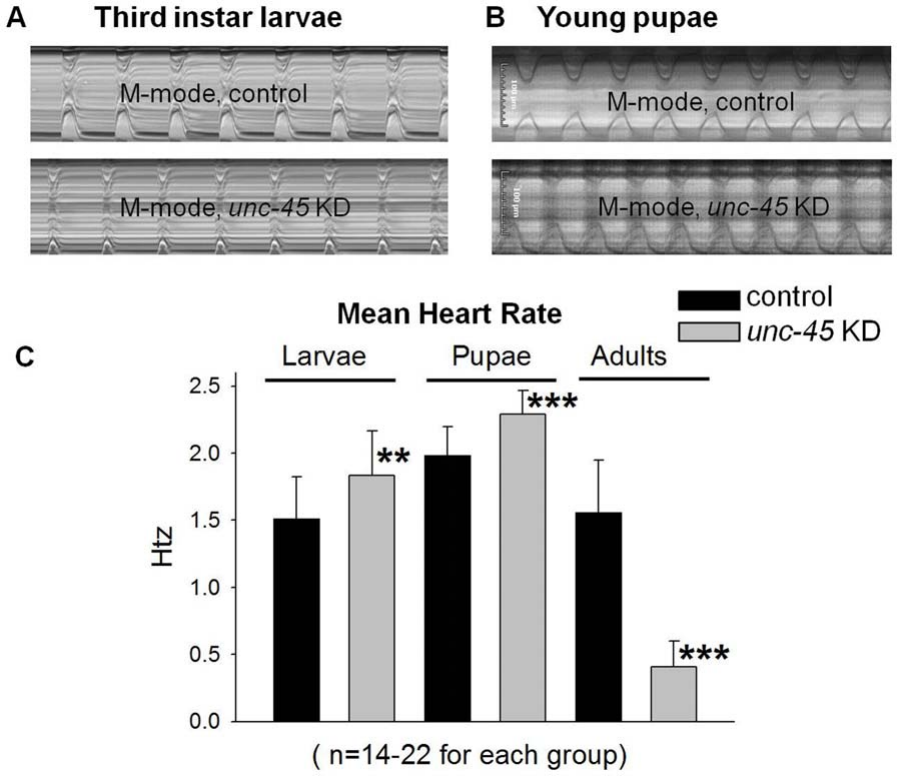
M-Mode analysis and quantification of heart rate in *unc-45* KD and control third instar larvae, young pupae and adult hearts. Cardiac parameters were determined as described in Materials and Methods. (**A, B**) M-modes (4 sec) from control (top) and *unc-45* KD (bottom) third instar larvae and young pupae (as described in Figure 1). (**C**) Mean heart rate of *unc-45* KD in third instar larvae and young pupae were significantly increased (tachycardia) compared to control. In contrast, heart rates were significantly reduced in adult hearts compared to controls. Heart rate data are shown as means ± SD; statistical significance was determined using a multivariate Student's t test (**  = p<0.01 and ***  =  p<0.001).

Since the *Hand*-Gal4 driver used to KD *unc-45* is expressed in the heart from embryogenesis through adulthood, results of manipulations using this driver do not allow us to distinguish whether *unc-45* is required during development for establishing normal heart function, or whether it is needed to maintain cardiac integrity during adulthood. Previous analyses demonstrated that during metamorphosis the *Drosophila* heart undergoes significant remodeling with major morphological and structural transformations [42]–[44]. A critical function of UNC-45 during cardiac metamorphosis is supported by transcriptome data indicating that *unc-45* mRNA expression is progressively up-regulated in the remodeling heart during metamorphosis [44]. Consequently, we wondered if a requirement for UNC-45 was critical during metamorphosis. To approach this question, we examined *Hand*>*unc-45-RNAi* hearts 1-4 days after eclosion and found that, as for hearts from 1-week or older *unc-45* KD flies, cardiac function was significantly compromised compared to age-matched controls (Figure S3A–C). Severe cardiac dysfunction was also observed as early as late pupal stages (similar to *unc-45* KD in the adults, as shown in Movie S1). In contrast, no major apparent cardiac defects were observed in *unc-45* KD hearts of third instar larvae and young (white) pupae regarding cardiac contractility compared to wild type. Cardiac contractility of control and *unc-45* KD young pupae is shown in Movie S2 and M-mode analysis of control and *unc-45* KD in third instar larvae and young pupae are shown in Figure 5A and B respectively. However, quantitative analyses of cardiac data obtained from both third instar larvae and young pupae revealed that *unc-45* KD hearts showed a small but significant increase in heart rate (tachycardia) compared to age-matched control (Figure 5C). This mild and possibly opposite phenotype contrasts with the severe heart defects seen with *unc-45* KD in adult hearts (Figure 5C). In addition to tachycardia, *unc-45* KD in third instar larvae heart resulted in mild but significant cardiac dilation, which is more evident after metamorphosis. As shown in Figure S4A and B both diastolic and systolic diameters of *unc-45* KD third instar larvae hearts were significantly larger compared to control hearts. Although *unc-45* KD third instar larvae hearts showed tachycardia, their cardiac performance (% FS) was depressed compared to control third instar larvae (Figure S4C), which confirmed an early manifestation of cardiac insufficiency and represents an attempt to compensate for the observed decrease in cardiac contractility/efficiency. We speculate that the continued requirement for UNC-45 during later stage metamorphosis results in more severe cardiac deficits that may not permit such compensatory changes in heart rate.

**Figure 6:**
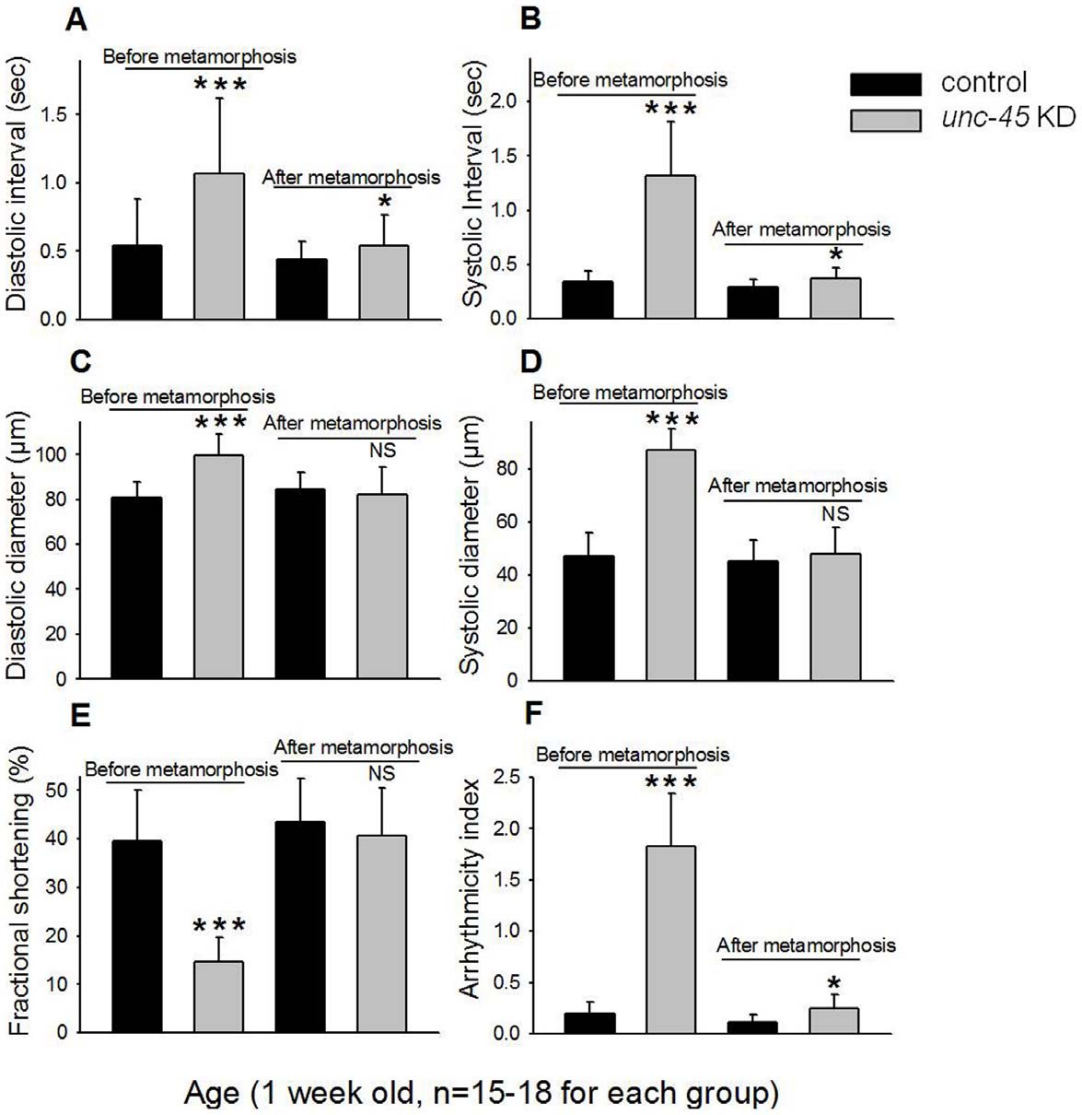
Requirement for UNC-45 during cardiac remodeling. (**A–F**) 1 week old adult hearts were analyzed after *unc-45* KD before or after metamorphosis. The left two bars show data from control and *unc-45* KD hearts when KD occurred prior to metamorphosis, i.e., starting at the third instar larval stage. The right two bars in each panel represent data from hearts when KD was performed after metamorphosis, i.e., in the adults. Data show mean values ± SD; statistical significance was determined using an unpaired Student's t test (***  =  p<0.001, *  =  p 0.05 and NS =  no statistical difference).

To further explore the timing of UNC-45 function during metamorphosis, we used the TARGET system [49], which allows regulated Gal4 expression via the temperature-sensitive Gal4 inhibitor, Gal80^ts^. Using the cardiac-specific *TinCΔ4*-Gal4 driver in conjunction with Gal80^ts^, we induced *unc-45* KD by shifting the temperature to 29°C in late third instar larval stage (before metamorphosis) or in young adults (after metamorphosis). Control flies were also exposed to the same temperature conditions. Conditional cardiac KD of *unc-45* with the *TinCΔ4*-Gal4; Gal80ts starting prior to metamorphosis resulted in a severe cardiac phenotype in adult hearts (Movie S3), similar to that obtained with *Hand*-Gal4 throughout life (Figure 2 and Movie S1). For example, 1 week old heart that had *unc-45* KD beginning before metamorphosis (third instar larval stage) showed prolonged systolic and diastolic intervals compared to controls (Figure 6A,B). In addition, such *unc-45* KD hearts were dilated (Figure 6C,D), their % FS was reduced (Figure 6E), and cardiac arrhythmias were increased (Figure 6F). Severe cardiac defects are also apparent in newly enclosed adults and late pupae when *unc-45* KD was carried out beginning at the young pupal stage instead of third instar larval stage (similar to Movie S3). These findings suggest that *unc-45* is required after embryogenesis and larval stages for proper establishment of heart structure and function.

In dramatic contrast to the above data, when *unc-45* was knocked down in the heart after metamorphosis (adult flies), dilation of the heart did not occur (Figure 6 and Movie S3); however, systolic and diastolic intervals were prolonged and arrhythmias were elevated (Figure 6A, B and F). Similar results were obtained when examining the hearts from older flies (as in Figure 6 and Movie S3). Since the heart phenotypes were much less dramatic when *unc-45* KD was initiated after eclosion, we conclude that optimal UNC-45 protein levels are particularly critical during remodeling of the adult heart and establishment of adult heart function during metamorphosis, however, UNC-45 is also required to some extent for cardiac maintenance later in adult life.

### Transgenic over-expression of UNC-45 rescues the cardiac phenotype in *unc-45* KD flies

**Figure 7:**
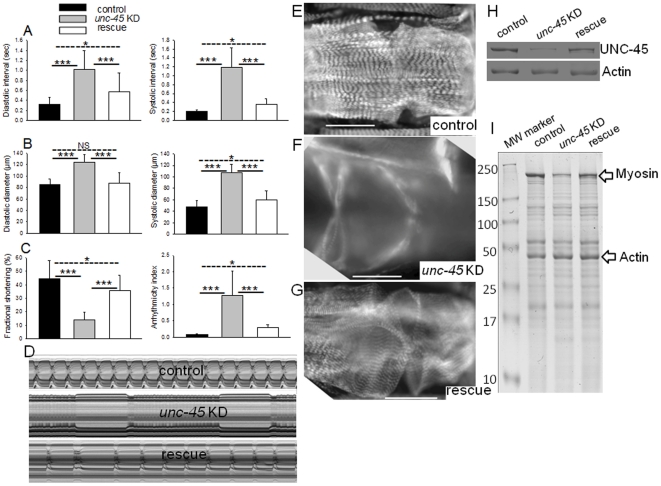
Transgenic over-expression of UNC-45 rescues defects associated with *unc-45* KD. (**A**) Assessment of cardiac diastolic and systolic intervals, (**B**) diastolic and systolic diameters, (**C**) % FS and cardiac arrhythmia, in 1 week control, *unc-45* KD and rescued old flies. Statistical difference of *unc-45* KD from control and rescued as well as between KD and rescued are represented as mean ± SD; where *** =  p<0.001, * =  p<0.05 and NS =  not significant). (**D**) M-Mode records (10 sec) from hearts of 1 week old control (top), *unc-45* KD (middle) and *unc-45* rescue (bottom). (**E-G**) myosin immunofluorescence micrographs of 1 week old control (top), *unc-45* KD (middle) and rescue (bottom), scale bar  = 50 µM. (**H**) Immunoblot (probed with UNC-45 and actin antibodies) and (**I**) SDS-PAGE shows myosin expression in control, *unc-45* KD and rescued fly hearts.

We tested whether transgenic over-expression of UNC-45 could rescue the *unc-45* KD cardiac phenotype. UNC-45 was over-expressed in the *unc-45* KD heart (Figure S5) and examination of all cardiac parameters showed significant improvement (Figure 7 and Figure S6). For example, diastolic and systolic intervals of 1 week old rescued hearts were significantly lower compared to *unc-45* KD hearts (Figure 7A). Cardiac dilation in the *unc-45* KD was partially rescued with transgenic over-expression of UNC-45; both diastolic and systolic diameters of hearts in rescue flies were significantly lower than those of *unc-45* KD hearts (Figure 7B). Cardiac efficiency in terms of % FS was greatly improved in rescue hearts (Figure 7C). Finally, the incidence of arrhythmias of rescued hearts was significantly reduced compared *unc-45* KD hearts (Figure 7C,D and Movie S4). Analysis of myofibril organization and myosin accumulation in control, KD and rescued hearts (Figure 7E–G) provided further evidence to indicate suppression of dilation and a partial rescue of myofibril disarray. Cardiac *unc-45* transgene expression restored UNC-45 and myosin protein accumulation in rescued hearts (Figure 7H,I). Finally, cardiac specific restoration of *unc-45* function also partially rescued the reduced life-span of *unc-45* KD flies, with maximal life-span being almost as long as controls (Figure S7). Note that *unc-45* RNAi targets *unc-45* transgene transcripts as well, which obviates complete phenotypic rescue.

Taken together, our data indicate that most of the cardiac defects resulting from *unc-45* inhibition were significantly improved by over-expression of an UNC-45 transgene. These results demonstrate that major structural and functional cardiac defects are induced by UNC-45 reduction, not by off-target RNAi effects. Using three different RNAi constructs and two different cardiac drivers we confirmed that the main cardiac defects are generated during metamorphosis, which indicates a critical requirement of UNC-45 in cardiac remodeling to give rise to a well-functioning adult heart.

## Discussion

Mutation of myosin or its loss from muscle fibers leads to several human diseases including cardiomyopathy and skeletal muscle myopathies [3]–[10]. Therefore maintaining myosin function is crucial for retaining cardiac and skeletal muscle performance. The assembly and folding of myosin, which is critical for myofibrillogenesis, is dependent on the chaperones UNC-45 and HSP-90 [22], [25], [32]. Previous *in vitro* and *in vivo* studies have shown that UNC-45 acts as a chaperone to assist myosin folding and/or accumulation [17], [20]–[23]. Furthermore, inhibition of *UNC-45B* expression by a morpholino antisense oligomer or mutation of *UNC-45B* in fish and frog embryos results in paralysis with skeletal muscle and cardiac dysfunction [22], [25], [32], [33]. Using the *Drosophila* model system, we recently showed that UNC-45 is expressed throughout development and that a null mutation of *unc-45* leads to embryonic lethality [17]. Consistently, KD of *unc-45* in all muscles also leads to a severe reduction in myosin content (Figure S1).

To elucidate a cardiac role for UNC-45 in the adult, we silenced UNC-45 expression in *Drosophila* heart, a model that allows for precise quantification of cardiac physiological parameters [8], [38]. In contrast to the *unc-45* KD in fish and frog embryos, KD in the remodeling fly heart did not cause complete paralysis, but drastically compromised function, reminiscent of cardiomyopathies caused by myosin mutations in flies or humans [3]–[8]. KD of *unc-45* in the *Drosophila* heart results in dramatic dilation as well as a significant reduction in cardiac output due to both a reduction in rate as well as reduced contractility. Consistent with a role in cardiac dilation, *unc-45* KD also caused cardiac myofibrillar disarray (Figure 3), similar to what is seen in skeletal muscles [22], [25], [32]. Although UNC-45 has not yet been implicated in vertebrate dilated cardiomyopathy, our unique observation of a dilated cardiomyopathy phenotype suggests that myosin chaperones are novel heart disease gene candidates to explore chaperone-based cardiomyopathy. Our findings may provide insight into understanding the role of UNC-45 in myosin-based cardiomyopathy, since we demonstrate here that the heart phenotype of *unc-45* deficient flies is closely mimicked by directly depleting the myosin content in the heart. This suggests that UNC-45 plays a crucial role in stabilizing myosin to prevent cardiomyopathy. It will be interesting to see if reduced function of UNC-45 in a mammalian model also causes myosin depletion and compromised heart function. Several chaperones have previously been shown to be associated with cardiac development and to exhibit enhanced expression in failing human hearts, possibly to combat the burden of misfolded proteins that can lead to cardiac dysfunction [12]–[14]. Furthermore, mutations of chaperones or their deficiency has indeed been associated with cardiomyopathies. For example, it is known that depletion of the mitochondrial chaperone HSP-40 and mutation of the titin-binding chaperone alpha B-crystallin in vertebrate heart lead to dilated cardiomyopathy [50], [51].

Because UNC-45 is known to act as a myosin chaperone, we tested the impact of *unc-45* KD on myosin expression in the heart and found a significant reduction (Figure 3). This suggests that *unc-45* plays a critical role in regulating the level of myosin by influencing the balance between synthesis and degradation. During adult stages when myosin synthesis is stabilized, UNC-45 function seems to be less important. Silencing of *unc-45* in zebrafish muscles showed reduced actin expression [22]. In contrast, actin levels in *unc-45* KD fly hearts and embryos were not significantly different from controls (Figures 3E, S-1 and S-2). Our results demonstrate that UNC-45 plays a role in the correct incorporation of myosin into sarcomeric structures, which is further supported by our finding that direct inhibition of *Mhc* expression reproduced the *unc-45* KD phenotype (Figure 4A–F). These results indicate that inhibition of *unc-45* expression interferes with appropriate myosin accumulation during cardiac remodeling, thus causing insufficient incorporation into cardiac sarcomeres, which in turn seems to cause the observed myofibril disorganization, as well as heart dilation and probably indirectly other cardiac physiological defects.

We showed that the cardiac defects we observe are likely the direct effect of reducing *unc-45* function, as opposed to off-target RNAi silencing. This is demonstrated by the significant rescue of the cardiac defects in two independent UNC-45 over-expression lines. In addition, UNC-45 transgenic over-expression enhanced myosin accumulation and extended life-span compared to *unc-45* KD flies. The fact that transgenic over-expression of UNC-45 resulted in only a partial rescue (Figure 7) is to be expected, since the *unc-45* RNAi silencing construct likely also targets the transgenic *unc-45*. This effect will be dose dependent, resulting therefore in only a partial rescue of physiological function, improved myofibril accumulation as well as increased myosin and UNC-45 expression compared to the *unc-45* KD (Figure 7).

The *Drosophila* heart undergoes major morphological and functional transformations during metamorphosis, which results in an almost complete remodeling from the larval heart to the adult heart [42]–[45]. During this process and without the addition of new cardiomyocytes, the CC is generated in the first and second abdominal segments [42], [44], [45]. The time of pupal remodeling of the heart appears to encompass the critical temporal window for UNC-45 function in the heart. This is supported by the finding that *unc-45* KD in pupae, prior to metamorphosis, resulted in severe cardiac defects in the adult heart but KD after metamorphosis only mildly affects heart structure and function. A role for UNC-45 during cardiac metamorphosis is also supported by a study that showed that cardiac *unc-45* mRNA levels are progressively up-regulated during metamorphosis [44]. Thus, we hypothesize that UNC-45 serves as a key chaperone to promote the folding and accumulation of myosin used in remodeling of the heart during metamorphosis. As with very young pupal hearts, we did not see severe contraction defects in third instar larval hearts. However, quantitative analysis of third instar larval hearts (Figure 5 and Figure S3) showed early manifestations of cardiac insufficiency such as tachycardia, cardiac dilation and reduced cardiac performance, which probably represent an attempt to compensate for a decrease in cardiac contractility/efficiency. This minimal effect in larvae is likely because the requirement of UNC-45 is also minimal in the larval stage. This is based on high-throughput expression data and quantitative RT-PCR data in all developmental stages that revealed minimal expression of *unc-45* RNA during larval stages compared to high levels of expression during metamorphosis (modENCODE Temporal Expression Data for FBgn0010812; http://flybase.org/reports/FBgn0010812.html and unpublished data from our lab). This differential expression may be true for the heart as well [44]. Secondly, it is possible that the myosin folding before metamorphosis is more dependent upon other chaperones such as Hsp-70 and Hsp-90 [11], [23]. Thirdly, it is possible that maternally-inherited UNC-45 and/or UNC-45 remaining after KD (as KD of *unc-45* is not null) are sufficient to maintain cardiac contractility in the larval or young pupal hearts. Based upon these data and our cardiac phenotype, we speculate that the continued requirement for UNC-45 during later stage metamorphosis results in more severe cardiac deficits that may not permit compensatory changes in heart rate seen in larvae and young pupae. Thus, UNC-45 is critical for folding and assembly of myosin in an almost complete remodeling from the larval heart to the adult heart.

Our study represents the first evidence of a role for a molecular chaperone in *Drosophila* cardiac structure and function. UNC-45 appears to be critical for myosin incorporation into sacomeres/myofibrils of the myocardium, as it is in skeletal muscles. Since this myosin chaperone is critical for maintaining the structural integrity of the cardiac contractile apparatus, it is possibly essential for human cardiac function and survival as well. Indeed, proteomic analysis of hearts from patients suffering from ischemic heart failure has detected increased levels of UNC-45, supporting the hypothesis that this chaperone may be important during human cardiac arrest [52], possibly in preventing myosin unfolding. Conversely, UNC-45 may play a role in a repair process by facilitating the incorporation of new myosin into damaged cardiomyocytes. Previous findings [25], [32], [33], combined with our study reveal that UNC-45 is critically required for heart development, remodeling and function, as KD or mutation leads to paralysis, lethality or severe cardiac dilation. Based on these findings we speculate that UNC-45 could play a role in childhood cardiac diseases and during maturation of the heart. Elucidating UNC-45 function in the heart will be crucial for understanding myosin-based cardiomyopathies and may provide a new target for therapeutic agents designed to reverse such debilitating human conditions.

## Materials and Methods

### *Drosophila* stocks and screening system

Two UAS-RNAi fly lines (construct IDs 9815 and 101311) for the *unc-45* (CG2708) gene were obtained from the Vienna *Drosophila* RNAi Center (VDRC). Each RNAi transgene was made with inverted repeat of an unc*-45* fragment, driven by the UAS-promoter as previously reported [48]. UAS-RNAi transgenic lines with insert in 2^nd^ and 3^rd^ chromosome (stock IDs 2708R-1 and 2708R-2) for *unc-45* were also obtained from the National Institute of Genetics Fly Stock Center (NIG), Tokyo, Japan. Myosin RNAi transgenes (CG17927) were also obtained from VDRC and NIG (construct IDs 1485 and 102402 from VDRC and stock ID 17927 R-1 from NIG). The cardiac tissue-specific *Hand*-Gal4 driver was gift from Eric Olsen [47] and the Gal4–Gal80 system driver (tub-Gal80-ts; TinCΔ4-Gal4) was a kind gift from Manfred Frasch [53]. The muscle specific driver 24B-Gal4 was obtained from Norbert Perimmon [46]. Transgenic *unc-45* lines were generated as recently described [17].

For RNAi silencing, *unc-45* RNAi males or virgin females were crossed to *Hand*-Gal4 flies and incubated at 25°C throughout development. Male and female F-1 progeny were separated and allowed to develop with food changes every third day. The temperature-sensitive Gal4–Gal80 system was used to control timing of RNAi KD (tub-Gal80-ts; TinCΔ4-Gal4) as previously reported [49]. Briefly, flies were crossed and progeny were raised at 25°C, KD was induced by raising the temperature to 29°C for the periods of time indicated in the text. Control flies included progeny of *w* ^1118^ flies crossed with *Hand*-Gal4 or tub-Gal80-ts; TinCΔ4-Gal4 or *w* ^1118^ flies crossed with each of the UNC-45 RNAi transgenic lines. Results from female progeny are reported here.

### Semi-intact *Drosophila* heart preparation, image analysis and structural studies

Semi-intact hearts were prepared as described previously [38], [54], [55]. Direct immersion optics were used in conjunction with a digital high-speed camera (up to 200 frames/sec, Hamamatsu EM-CCD) to record movies of contraction movements using the image capture software HC Image (Hamamatsu Corp.). Movie analysis was carried out using our semi-automatic heartbeat analysis software which quantified heart period, diastolic and systolic diameters, diastolic and systolic intervals, cardiac rhythmicity, fractional shortening and produced the M-mode records [38], [54], [55]. Fluorescence imaging of *Drosophila* heart tubes with myosin antibody or fluorescently-labeled phalloidin was carried out using an Apotome Imager Z1 (Zeiss) and an AxioCam MRm (Zeiss) microscope as previously described [40], [56]. Fluorescent probes Alexa555-phalloidin (Invitrogen, Carlsbad, CA) and goat-anti-rabbit-Cy5 (Chemicon, Temecula, CA) were used. Semi-intact third instar larvae hearts were exposed by dissection and analyzed as for adult hearts. Analyses of young (white) pupae hearts were carried out as for the adult heart, but without dissection.

### Biochemical analysis

For analysis of UNC-45 and myosin expression, specimens were collected and protein was extracted with Laemmli sample buffer (Bio-Rad, Hercules, CA) containing 200 mM β-mercaptoethanol [57]. Extracted samples from equal numbers of hearts, indirect flight muscles or embryos were separated by 10% SDS-PAGE and immunoblotting was carried out using a rabbit anti-*Drosophila* UNC-45 antibody as recently described [17] and mouse anti β-actin antibody (Sigma, St. Louis, MO). Each primary antibody (1:1000 dilution) was incubated with the membrane for 4 hours to overnight at 4°C and washed three times in PBST at room temperature. Incubation of the membranes with goat-anti-rabbit HRP (UNC-45) or goat-anti-mouse-HRP (actin) were carried out for 4 hours to overnight as previously described [17]. Secondary antibodies (BioRad, Hercules, CA) were added at 1:2000 dilution for 2 hours at room temperature. HRP conjugated antibodies were visualized by incubating membranes with SuperSignal West Pico Chemiluminscent substrate (Pierce). For myosin expression, extraction of specimens was carried and evaluated by analysis of protein samples separated on 10% SDS-PAGE and stained with either Coomassie blue or Coomassie fluoro (Invitrogen, Carlsbad, CA).

### Transgenic rescue of *unc-45* KD hearts

Transgenic rescue of the *unc-45* KD was carried out using standard genetic techniques as outlined in Figure S5. UNC-45 transgenes used in this study were previously tested to rescue an *unc-45* mutant [17]. Both VDRC and NIG *unc-45* RNAi stocks were rescued with transgenic over-expression of UNC-45. Cardiac physiological, structural and biochemical analyses of the rescued hearts were carried out as reported for control and *unc-45* KD hearts.

## Supporting Information

Figure S1 **RNAi KD of** ***unc-45*** **with the 24B-Gal4 driver and its impact on myosin expression.** (**A**) Immunoblot analysis of unc-45 expression (top) in 20 h old embryos from control (24B-Gal4/+) and *unc-45* KD (24B-Gal4 >*unc-45*RNAi-1 (NIG) and 24B-Gal4 > unc45 RNAi-2 (VDRC)) flies. UNC-45 expression was reduced significantly (∼70-80%) in the KD embryos. (**B**) Myosin content was reduced significantly in the *unc-45* KD embryos (as analyzed by SDS-PAGE), however actin content appears to be similar for all groups. Each lane represents the total extracted protein from 20 embryos.
(TIF)

Figure S2 **Knock down of** ***unc-45*** **results in significant reduction in both myosin accumulation and myofibrillar organization.** Immunofluorescence micrographs of cardiac tubes from 1 week old flies are shown. (**A, B**) Hearts from controls and (**C, D**) *unc-45* KD flies were probed with antibody against muscle myosin and phalloidin respectively as described in the main test. Control cardiac tubes show typical spiral myofibrillar arrangements within the cardiomyocytes (A, B). Myofibrillar organization is completely disrupted in *unc-45* KD with loss of most myosin-containing myofibrils and significant dilation (C). Remarkably, even with minimal myosin present, myofibrils still form, albeit in a considerably disorganized fashion, as seen by probing with labeled phalloidin, which binds to filamentous actin (D). Myofibrils within cardiomyocytes are shown with blue arrows in A, B and D. KD of *unc-45* leads to loss of most myosin-containing myofibrils in cardiac muscle whereas longitudinal ventral muscle myofibrils (white arrow in C) remain unaffected. All images were taken at 25X magnification.
(TIF)

Figure S3 **Cardiac defects associated with** ***unc-45*** **KD in 4-day old adults.** (**A**) Hearts (4-day old) from *unc-45* KD flies show prolonged diastolic and systolic intervals compared to control hearts. (**B**) Diastolic and systolic diameters of the KD hearts were significantly higher compared to age-matched control hearts. (**C**) Cardiac contractility (% fractional shortening) of the *unc-45* KD hearts was significantly reduced and significant cardiac arrhythmia was observed. Mean values ± SD are shown. Statistical differences between control and *unc-45* KD hearts were determined using an unpaired Student's t test (*** =  p<0.001).
(TIF)

Figure S4 **Cardiac defects associated with** ***unc-45*** **KD in third instar larvae.** Comparison of cardiac diameter (diastolic and systolic (**A**) and (**B**), respectively) and cardiac efficiency (% fractional shortening, (**C**)) in control and *unc-45* KD hearts from third instar larvae. Both diastolic and systolic cardiac diameters were significantly increased in *unc-45* KD larvae hearts. Cardiac performance of *unc-45* KD third instar larvae were significantly reduced compared to age matched controls. Statistical differences between control and *unc-45* KD hearts were determined using an unpaired Student's t test (* =  p<0.05).
(TIF)

Figure S5 **Scheme for transgenic over-expression of unc-45 to rescue defects associated with** ***unc-45*** **KD.** Genetic crosses using multiple balancers were carried out to rescue cardiac phenotypes associated with *unc-45* KD. *unc-45* RNAi and a cardiac driver (*Hand*-Gal4) transgenes are inserted in the second chromosome and the transgenic *unc-45* gene described in the scheme is inserted in the fourth chromosome. Transgenic *unc-45* inserted in the X-chromosome was also used to rescue the cardiac phenotype associated with *unc-45* KD.
(TIF)

Figure S6 **Transgenic over-expression of** ***unc-45*** **partially rescues defects associated with** ***unc-45*** **KD.** As for one week-old flies (main text, Fig. 7 A-C), cardiac physiological parameters of 2 and 3 week-old (n =  27-35 for each group) *unc-45* KD hearts were significantly improved by over-expression of *unc-45* compared to same age controls. Statistical differences between control and *unc-45* KD hearts were determined using an unpaired Student's t test (*** =  p<0.001; * =  p<0.05).
(TIF)

Figure S7 **Transgenic over-expression of** ***unc-45*** **partially rescues lethality associated with** ***unc-45*** **KD.** Cardiac-specific KD of *unc-45* results in a decrease in mean life span (over 80% of flies are dead within 3 weeks compared to 100% survival for the same time period in the control). This reduced lifespan was partially rescued by transgenic over-expression of UNC-45, as only ∼15% of flies are dead in 3 weeks. The average of a total of 250 flies from three experiments was determined for each group.
(TIF)

Movie S1 **Control and** ***unc-45*** **KD adult beating hearts.** Representative movies of 1 week old *Drosophila* hearts; the first clip (Control, first 10 sec) shows a typical regularly beating heart. The following clip (*unc-45* KD, second 10 sec) shows the irregular beating pattern observed in response to *unc-45* KD in the heart. This *unc-45* KD heart also exhibits the significant dilation and the reduction in contractility that is typical of these KD hearts.
(MOV)

Movie S2 **Control and** ***unc-45*** **KD white pupae beating hearts.** Movie clips of white pupae (young) showing contractions in control hearts (first clip, 10 sec) and *unc-45* KD hearts (second clip, 10 sec). Note increased heart rate in response to *unc-45* KD.
(MOV)

Movie S3 **Adult beating hearts that had** ***unc-45*** **KD before or after metamorphosis.** Movie clip of 1 week old adult fly hearts where *unc-45* KD was carried out either before (”Before Metamorphosis”, first 10s) or after metamorphosis (following 10s) using the TARGET system and the heart specific TinCΔ4-Gal4 driver.
(MOV)

Movie S4 **Control,** ***unc-45*** **KD and rescued adult beating hearts.** Movies of control (first clip, 10 sec), *unc-45* KD (middle clip, 10 sec) and *unc-45* KD rescued with transgenic over-expression of UNC-45 (last clip, 10s). The compromised beating pattern and dilation seen in the *unc-45 KD* are greatly reduced in the rescued heart.
(MOV)
