# Supplementary figures and images for: The UNC-45 Chaperone Is Critical for Establishing Myosin-Based Myofibrillar Organization and Cardiac Contractility in the Drosophila Heart Model
Source: PLoS One. 2011 Jul 25;6(7):e22579. doi: 10.1371/journal.pone.0022579 (PMC3143160; doi:10.1371/journal.pone.0022579)

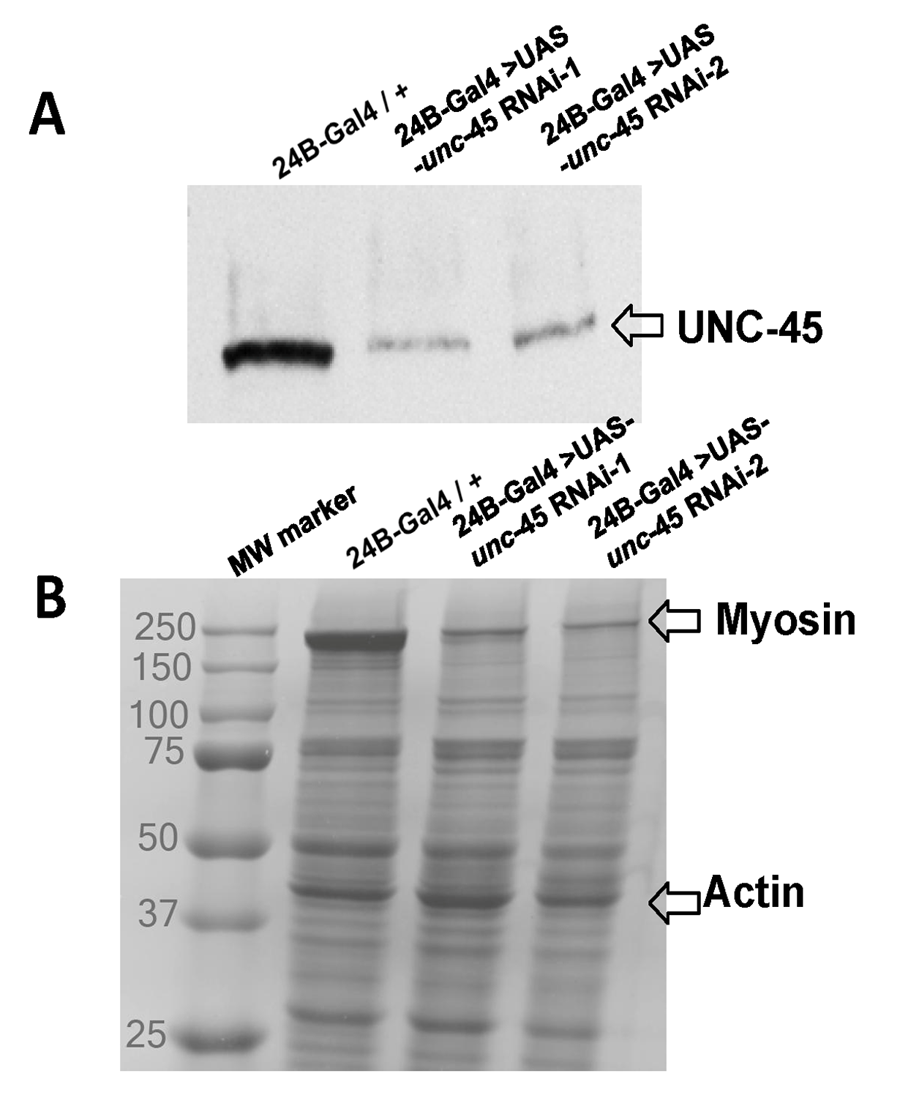

Supplement: Figure S1 — RNAi KD of unc-45 with the 24B-Gal4 driver and its impact on myosin expression. (A) Immunoblot analysis of unc-45 expression (top) in 20 h old embryos from control (24B-Gal4/+) and unc-45 KD (24B-Gal4 >unc-45RNAi-1 (NIG) and 24B-Gal4 > unc45 RNAi-2 (VDRC)) flies. UNC-45 expression was reduced significantly (∼70-80%) in the KD embryos. (B) Myosin content was reduced significantly in the unc-45 KD embryos (as analyzed by SDS-PAGE), however actin content appears to be similar for all groups. Each lane represents the total extracted protein from 20 embryos. (TIF) [file pone.0022579.s001.tif]

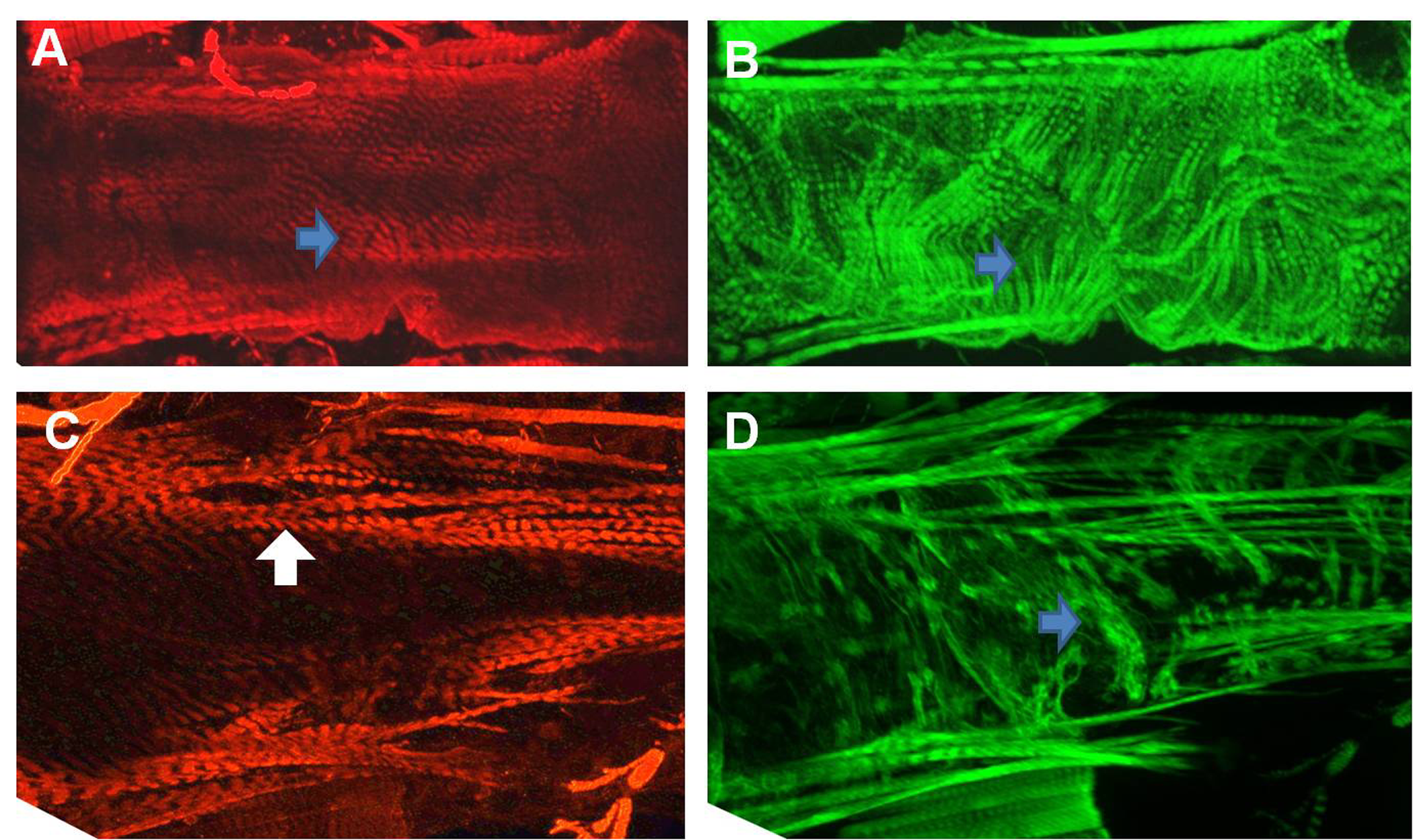

Supplement: Figure S2 — Knock down of unc-45 results in significant reduction in both myosin accumulation and myofibrillar organization. Immunofluorescence micrographs of cardiac tubes from 1 week old flies are shown. (A, B) Hearts from controls and (C, D) unc-45 KD flies were probed with antibody against muscle myosin and phalloidin respectively as described in the main test. Control cardiac tubes show typical spiral myofibrillar arrangements within the cardiomyocytes (A, B). Myofibrillar organization is completely disrupted in unc-45 KD with loss of most myosin-containing myofibrils and significant dilation (C). Remarkably, even with minimal myosin present, myofibrils still form, albeit in a considerably disorganized fashion, as seen by probing with labeled phalloidin, which binds to filamentous actin (D). Myofibrils within cardiomyocytes are shown with blue arrows in A, B and D. KD of unc-45 leads to loss of most myosin-containing myofibrils in cardiac muscle whereas longitudinal ventral muscle myofibrils (white arrow in C) remain unaffected. All images were taken at 25X magnification. (TIF) [file pone.0022579.s002.tif]

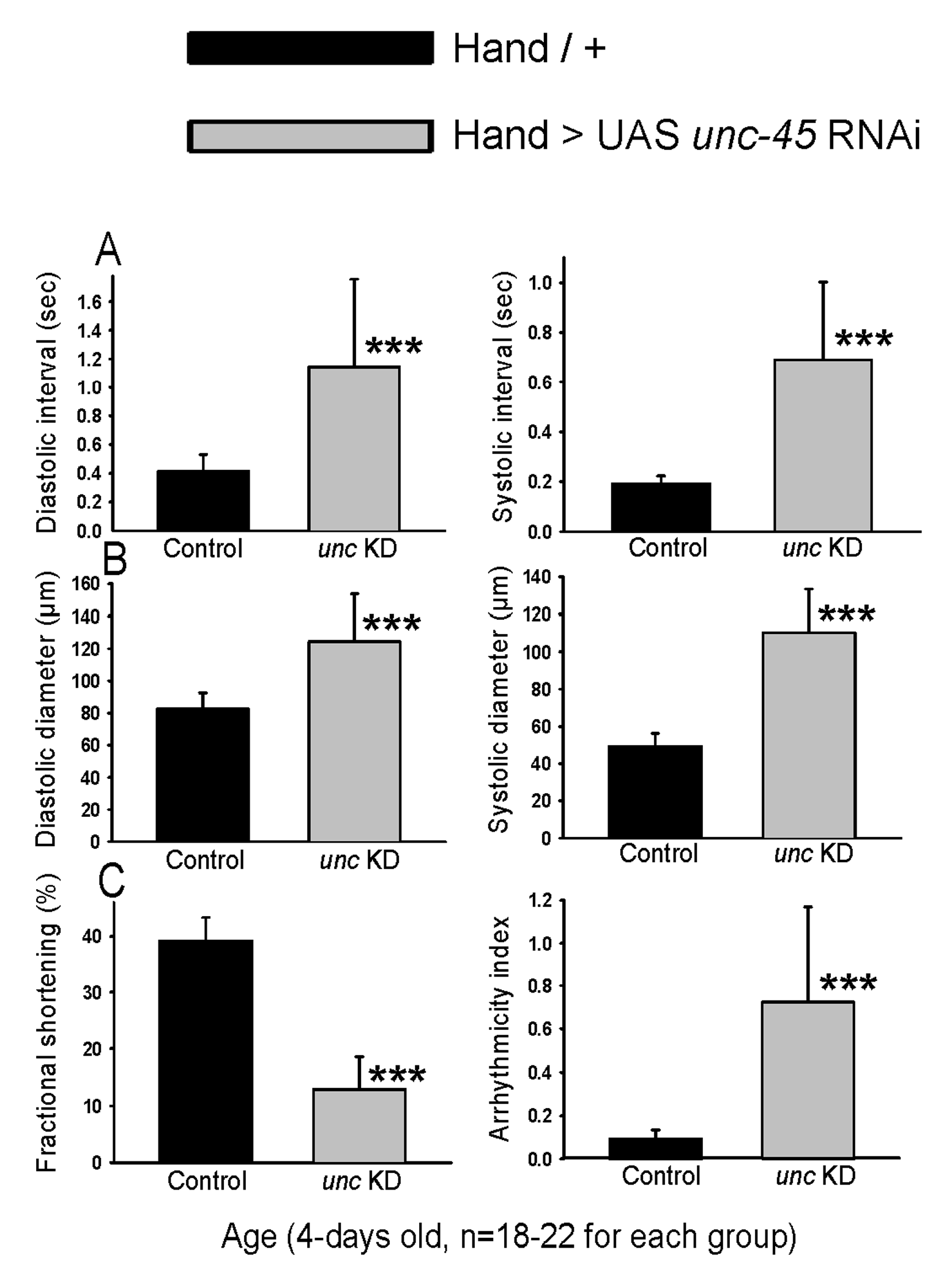

Supplement: Figure S3 — Cardiac defects associated with unc-45 KD in 4-day old adults. (A) Hearts (4-day old) from unc-45 KD flies show prolonged diastolic and systolic intervals compared to control hearts. (B) Diastolic and systolic diameters of the KD hearts were significantly higher compared to age-matched control hearts. (C) Cardiac contractility (% fractional shortening) of the unc-45 KD hearts was significantly reduced and significant cardiac arrhythmia was observed. Mean values ± SD are shown. Statistical differences between control and unc-45 KD hearts were determined using an unpaired Student's t test (*** = p<0.001). (TIF) [file pone.0022579.s003.tif]

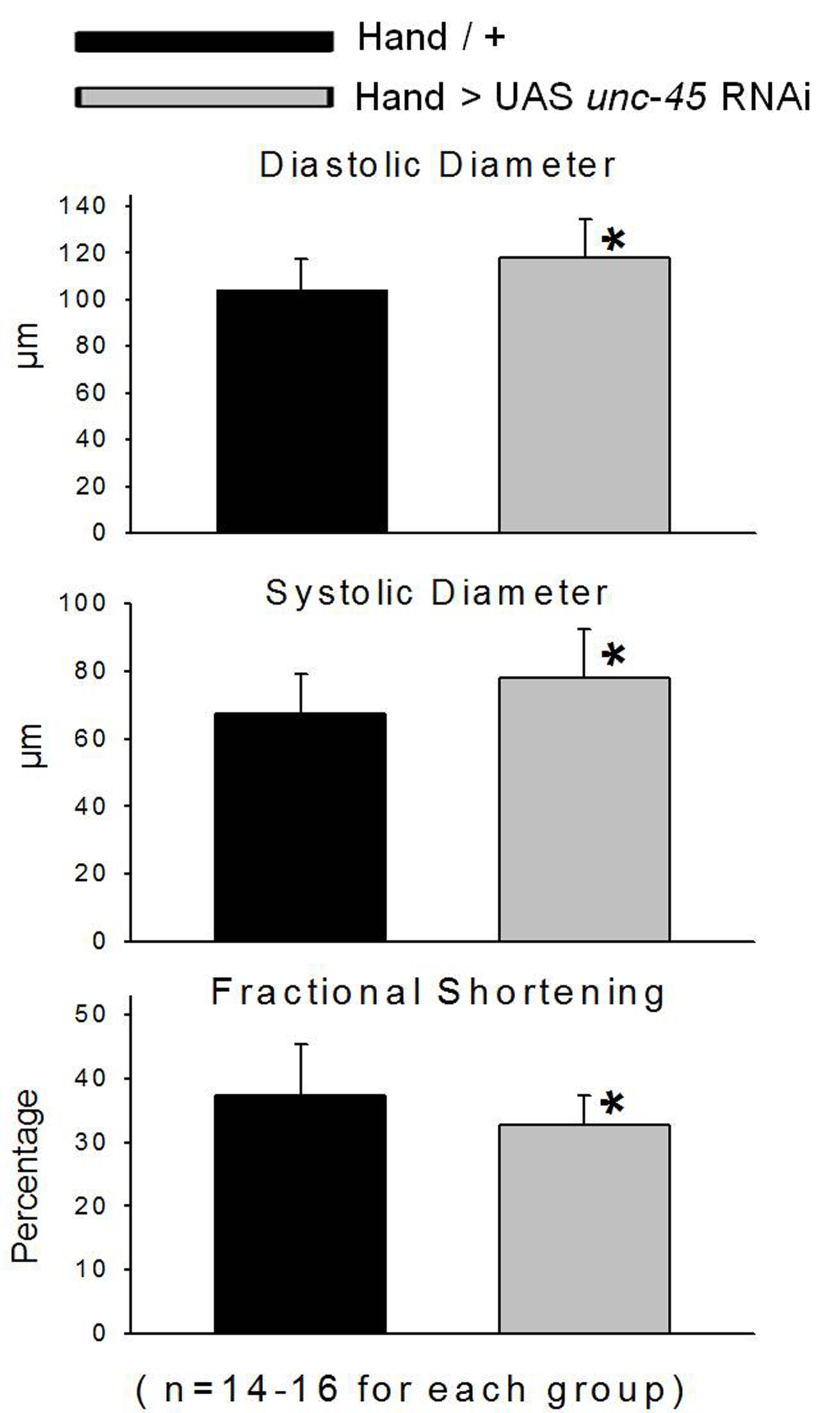

Supplement: Figure S4 — Cardiac defects associated with unc-45 KD in third instar larvae. Comparison of cardiac diameter (diastolic and systolic (A) and (B), respectively) and cardiac efficiency (% fractional shortening, (C)) in control and unc-45 KD hearts from third instar larvae. Both diastolic and systolic cardiac diameters were significantly increased in unc-45 KD larvae hearts. Cardiac performance of unc-45 KD third instar larvae were significantly reduced compared to age matched controls. Statistical differences between control and unc-45 KD hearts were determined using an unpaired Student's t test (* = p<0.05). (TIF) [file pone.0022579.s004.tif]

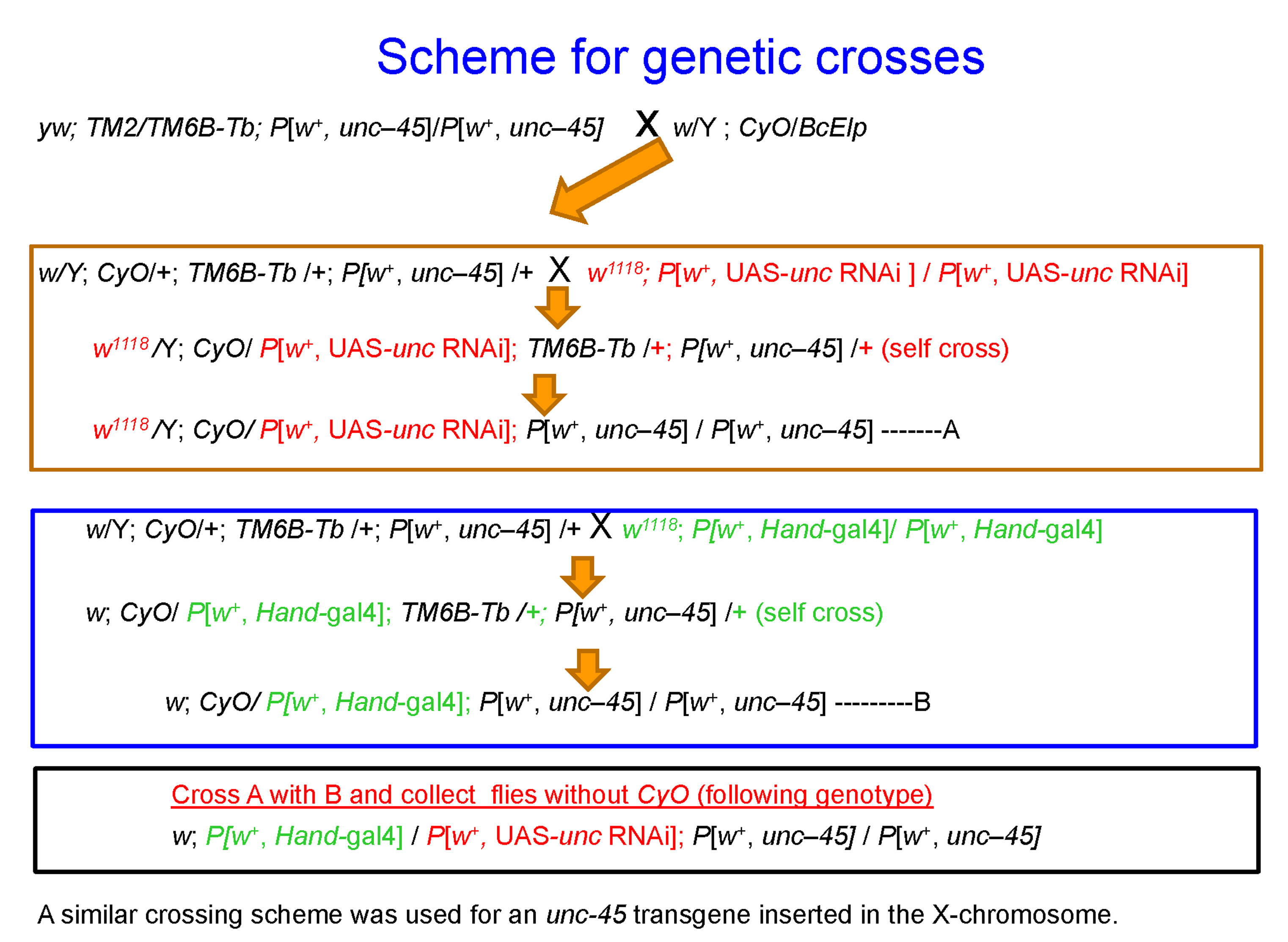

Supplement: Figure S5 — Scheme for transgenic over-expression of unc-45 to rescue defects associated with unc-45 KD. Genetic crosses using multiple balancers were carried out to rescue cardiac phenotypes associated with unc-45 KD. unc-45 RNAi and a cardiac driver (Hand-Gal4) transgenes are inserted in the second chromosome and the transgenic unc-45 gene described in the scheme is inserted in the fourth chromosome. Transgenic unc-45 inserted in the X-chromosome was also used to rescue the cardiac phenotype associated with unc-45 KD. (TIF) [file pone.0022579.s005.tif]

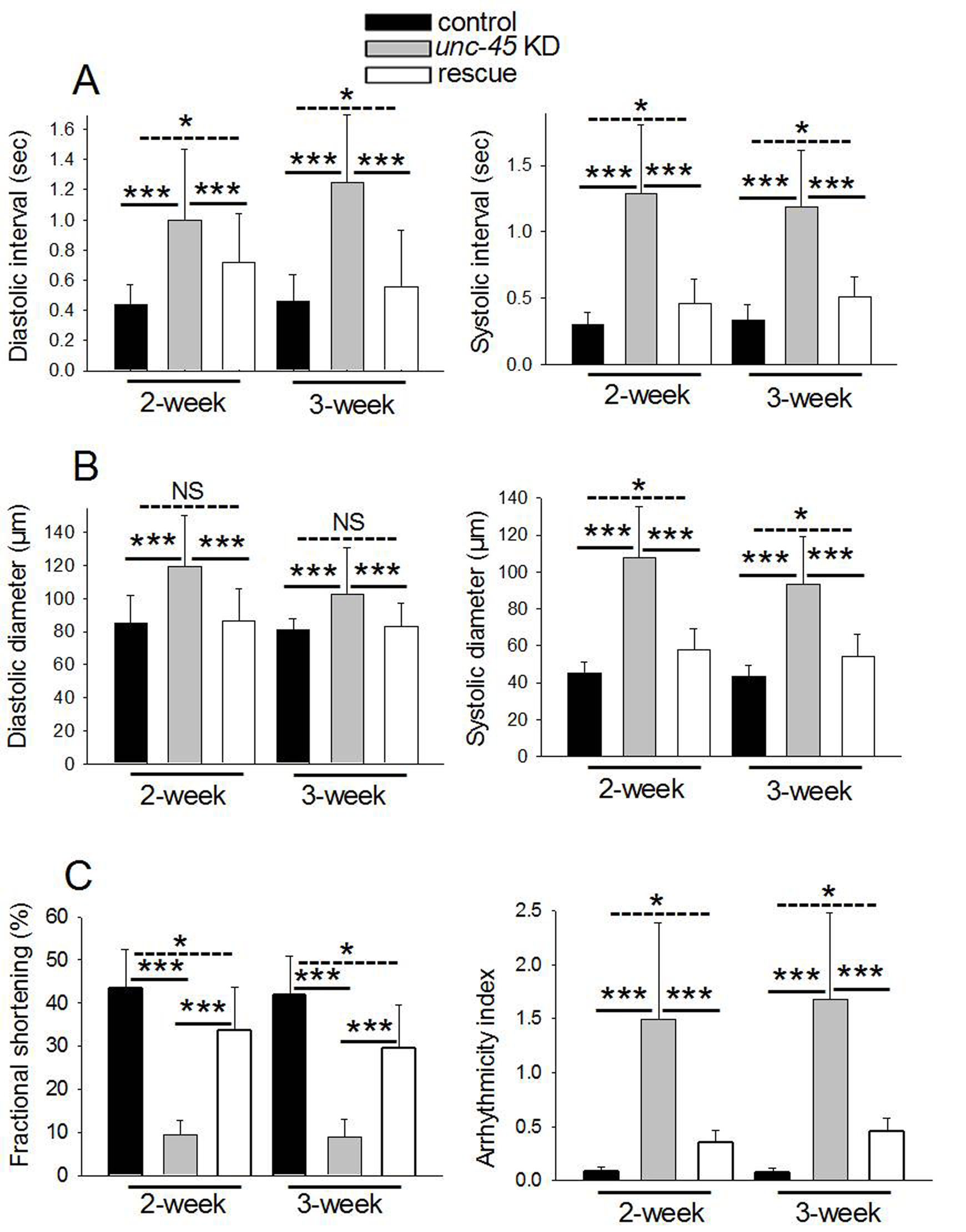

Supplement: Figure S6 — Transgenic over-expression of unc-45 partially rescues defects associated with unc-45 KD. As for one week-old flies (main text, Fig. 7 A-C), cardiac physiological parameters of 2 and 3 week-old (n = 27-35 for each group) unc-45 KD hearts were significantly improved by over-expression of unc-45 compared to same age controls. Statistical differences between control and unc-45 KD hearts were determined using an unpaired Student's t test (*** = p<0.001; * = p<0.05). (TIF) [file pone.0022579.s006.tif]

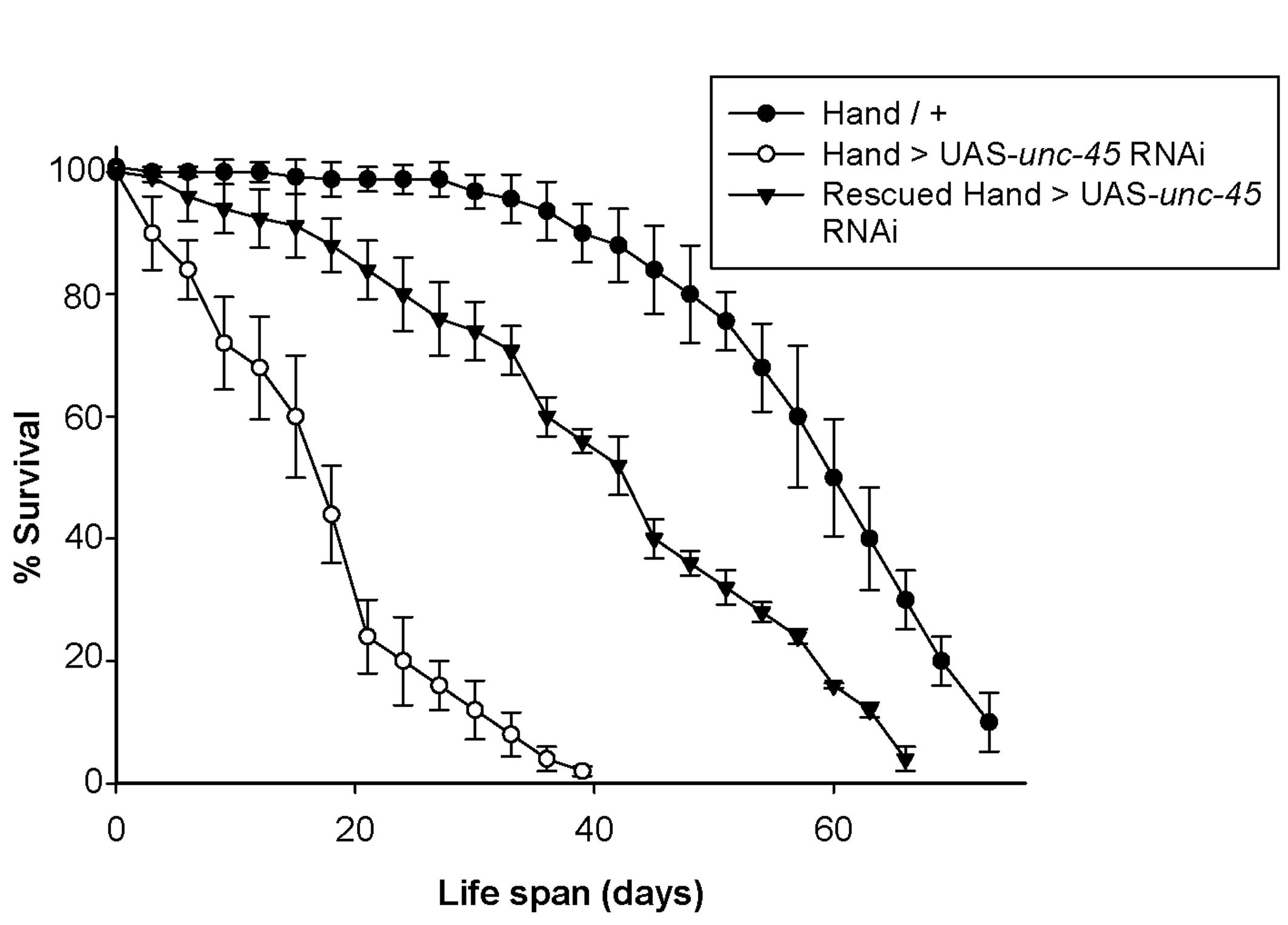

Supplement: Figure S7 — Transgenic over-expression of unc-45 partially rescues lethality associated with unc-45 KD. Cardiac-specific KD of unc-45 results in a decrease in mean life span (over 80% of flies are dead within 3 weeks compared to 100% survival for the same time period in the control). This reduced lifespan was partially rescued by transgenic over-expression of UNC-45, as only ∼15% of flies are dead in 3 weeks. The average of a total of 250 flies from three experiments was determined for each group. (TIF) [file pone.0022579.s007.tif]
